# Fibrin as a Tissue Adhesive and Scaffold with an Angiogenic Agent (FGF-1) to Enhance Burn Graft Healing In Vivo and Clinically

**DOI:** 10.3390/jfb9040068

**Published:** 2018-11-26

**Authors:** Dale S. Feldman, Scott Osborne

**Affiliations:** Department of Biomedical Engineering, University of Alabama at Birmingham, Birmingham, AL 35294, USA; 357dale@gmail.com

**Keywords:** fibrin tissue adhesive, fibrin scaffold, fibroblast growth factor-1, wound healing, burn graft healing

## Abstract

There is a need for a strategy to reduce scarring in meshed skin graft healing leading to a better cosmetic result without a significant increase in cost. The strategy in this paper is to increase the closure rate of a meshed skin graft to reduce scarring, which should also decrease the infection rate. Specifically, we used fibrin glue to attach all parts of the graft to the wound bed and added in an angiogenic growth factor and made the fibrin porous to further help the growth of blood vessels from the wound bed into the graft. There was a 10-day animal study and a one-month clinical study. Neither making the fibrin porous or adding an angiogenic agent (i.e., fibroblast growth factor-1 (FGF-1)) seemed to make a significant improvement in vivo or clinically. The use of fibrin on a meshed skin graft appears to speed up the regenerative healing rate leading to less scarring in the holes in the mesh. It appears to shorten the healing time by five days and keep the tissue stiffness close to normal levels vs. the doubling of the stiffness by the controls. A larger clinical study, however, is needed to definitively prove this benefit as well as the mechanism for this improvement.

## 1. Introduction

Burns as a result of chemical, thermal, or electrical injury are common, with two million people treated annually in the United States [1,2]. Of these, 100,000 people are hospitalized at almost $75,000 per admission, while nearly 4000 die [2,3,4]. In recent years, better infection control, better surgical techniques, and development of skin substitutes have reduced the death rate by over half from 30 years ago [4,5,6,7,8,9,10,11,12,13,14,15,16,17,18,19,20,21,22]. Now, the critical needs are to not only get better in these three areas, but also to have better assessment techniques, faster wound closure times, and to improve the quality of the new tissues formed [23]. Speed of wound closure can have an effect on cost of treatment, infection rate, and quality of tissue [23,24]. The faster a wound closes, the lesser the risk of infection, and in general, the lower the cost [23,24]. Further, it has been shown that for many wounds, in particular burn wounds, the faster the healing rate, the less the wound contraction rate, which leads to less scarring [24,25,26]. Scarring in burn wounds can lead to cosmetic and functional issues [24,26]. One of the biggest problems is stiffness of the healed burn wound, which tends to scar particularly with meshed skin grafts, and requires over a year of rehabilitation to return the skin stiffness close to the native skin [24,26]. The need, therefore, is a strategy to reduce scarring in meshed skin graft healing leading to a better cosmetic result and a reduction in the cost and time of rehabilitation to restore function. The strategy in this paper is to increase the closure rate of a meshed skin graft to reduce scarring, which should also decrease the infection rate.

For full-thickness burns, the standard is to use autologous skin grafts [5]. For severe burns, however, which cover a high percentage of a patient’s body, allografts or tissue-engineered skin are needed. In most cases, surgeons use autologous meshed grafts, which are typically meshed to increase the area covered [5]. This allows more body surface area to be covered in a shorter amount of time, thereby reducing the chances of infection and enhancing efforts to restore homeostasis [5,6,7,8]. Because of the cosmetic and functional issues, unmeshed skin is used if possible on certain areas of the body such as the face or hands [5].

Synthetic sutures or staples placed at the wound edges are typically used to fix the meshed skin grafts, which are removed after the graft takes [5,8,11]. Furthermore, fixation and survival of skin grafts are related to the re-establishment of an adequate circulation [5,7,8,24]. This is another reason grafts are meshed; to avoid fluid collection under the graft making it more difficult for blood vessels from the wound bed to grow into or reattach to the old blood vessels in the skin graft [7,8,24].

The two strategies employed here are to use fibrin glue to attach all parts of the graft to the wound bed and add in an angiogenic growth factor to further help the growth of blood vessels from the wound bed into the graft. For skin graft healing, the first phase, occurring within 24 h of transplantation, has been termed “the stage of plasmatic imbibition” where the wound bed attempts to adhere to the graft through formation of a fibrin scaffold [6,8,10,27]. Subsequently, the graft swells with exudate, thus providing access to nutrients and preserving the patency of existing blood vessels within the graft [27]. Forty-eight to seventy-two hours after surgery, anastomosis formation occurs, followed by blood vessel budding and neovascularization [6,27]. During this period, ingrowth of fibroblasts, fibrocytes, and other cells occurs in attempt to incorporate the graft [27]. The graft, however, must be securely attached to the wound bed for this to occur [28]. Full blood and lymphatic circulation are usually restored in 4–7 days [6,27]. Sensory nerve regeneration begins 2–4 weeks after transplantation; full function may not return for many months [6,27]. Failure to establish a satisfactory blood supply between the graft and vascular bed will lead to graft loss when this fails to occur [6,27].

Fibrin tissue adhesives (FTAs) have become popular over the last twenty years as a method of surgical fixation [24]. Although the hemostatic properties of fibrin have been known for nearly one hundred years, development into an adhesive has progressed slowly [24]. Part of the issue was inadequate concentrations of fibrinogen reducing bonding strength, which led to premature repair failures [24]. It was not until the 1970s that improving technologies allowed for better separation of plasma components and the ability to obtain higher concentrations of fibrinogen [29]. Subsequently, FTAs have found use in diverse fields such as pediatrics, otorhinolaryngology, and burn care [24,28,29,30]. The use of FTAs with skin grafts has showed decreased wound contraction and good adhesion in areas difficult to immobilize [28,29], since fibrin is the normal mode of graft attachment to the wound bed [6,27]. Although studies have shown the ability of fibrin glue to help reduce contraction, these studies were qualitative and did not fully show the mechanisms involved in animal models or clinically.

Fibroblast growth factor-1 (FGF-1) is an angiogenic factor that has previously been incorporated into a degradable fibrin scaffold [31]. As enzymes degrade the fibrin clot, FGF-1 is released and enhances the angiogenic response [32]. Porous fibrin systems have been shown to increase angiogenic and fibroblastic response over controls and non-porous systems [31].

Therefore, the goal of this study was to increase the healing rate of an autologous meshed skin graft in order to make a significant difference in scar formation. This should translate to improvement in both cosmetic and functional clinical performance, as well as reduce the overall cost of the procedure. Specifically, the study was designed to use these systems in both an animal model and clinically. The intent was to not only quantify the benefit for clinical performance, but also quantify the changes in bioprocesses to help determine the mechanisms for any benefits seen. Healing of a graft is both the percentage area of the graft, which survives (graft take), as well as the filling in of the holes of the mesh (healing rate).

In the animal model, the performance measures were tissue stiffness and graft take percent at both three and ten days. To better understand the reason for the differences in performance, the number of cells and blood vessels were determined at each time period from three different regions of the wound.

## 2. Materials and Methods

### 2.1. Materials

#### 2.1.1. Fibrin Tissue Adhesive Components

Lyophilized fibrinogen from rabbit plasma (Sigma Chemical Company, St. Louis, MO, USA) was dissolved in a 50 mM histidine/150 mM NaCl buffer, at a pH of 7.4, to obtain a fibrinogen concentration of 60 mg/mL. Lyophilized bovine thrombin (Sigma, Suwanee, GA, USA) was dissolved in a 0.40 M CaCl_2_ solution to obtain a concentration of 2000 U/mL. This solution was then diluted with a thrombin diluent comprised of Earle’s medium (Biowest, San Marcus, TX, USA) in 40 mM CaCl_2_. Poly(ethylene oxide) (PEO) (Aldrich Chemical Company, Inc., Milwaukee, WI, USA) of 2,000,000 MW was screened using Tetko Precision Screening Media (Tetko, Inc., Briarcliff Manor, NY, USA) to obtain 170–240 µm particles. The PEO was then partitioned into 8 and 16 mg portions and stored at −20° C in FISHER brand 1.5 mL microcentrifuge tubes (Fisher Scientific, Pittsburgh, PA, USA).

#### 2.1.2. FGF-1

Heparin-sepharose affinity chromatography was utilized to purify FGF-1 recombinantly produced using E. coli under control of the trp-lac operon. The growth factor was stored at 4 °C at a concentration of 6000 µg/mL. Fibroblast growth factor-1was added to the fibrinogen solution to give a total concentration of 16 µg/mL.

### 2.2. Methods

#### 2.2.1. Surgical Techniques

Prior to the beginning of the procedure, each of the 10 White New Zealand rabbits, 2.6–3.4 kg, were anesthetized intramuscularly (IM) with a solution of ketamine, at a dose of 25–37 mg/kg and xylazine, at a dose of 5 mg/kg. The hair on the dorsum of each rabbit was clipped and carefully shaved with a surgical prep blade. The dorsal surface of the rabbit was then locally anesthetized with a solution of lidocaine and then scrubbed with Betadine Surgical Scrub (Aveco, Fort Dodge, IA, USA). Supplemental doses of ketamine were administered intramuscularly at 15–20 mg/kg as needed.

Using a Zimmer Air Powered Dermatome (Model #8801-01, Zimmer Patient Care Division, Dover, OH, USA) with medical grade dry nitrogen (Airco, Bessemer, AL, USA), two 2″ wide and 0.010-inch thick skin grafts were harvested from the lateral aspects of the dorsum. The grafts were then enlarged with a 3:1 mesher (Brennan, North Bergen, NJ, USA) and placed temporarily in sterile saline. Five 0.75″ × 0.75″ full thickness defects were made within the donor site areas. Sections of graft were then measured and trimmed to fit each defect. Subsequently, one of five treatment modalities were applied to adhere grafts to each wound: 4-0 proline sutures as control (S); fibrin glue (F); fibrin glue with FGF-1 (FW); porous fibrin glue (PF); or porous fibrin glue with FGF-1 (PFW). The defects were then covered with Tegaderm and the rabbit was wrapped with a Stockinette bandage (Johnson and Johnson, New Brunswick, NJ, USA) and a custom rabbit jacket (Alice King Chatham Medical Arts, Los Angeles, CA, USA).

The fibrin tissue adhesive components were applied stepwise via 1cc Tuberculin Syringes with 20G1 PrecisionGlide Needles (Bectin Dickinson and Company, Rutherford, NJ, USA). For the non-porous system, two drops of the thrombin solution (0.013 mL) was applied to the wound bed and spread to obtain an even distribution. Two drops of the fibrinogen solution (0.02 mL) were then added and mixed rapidly, yet thoroughly, and allowed to sit for 5 min. The graft was then placed upon the wound bed and spread to cover the defect. The graft was then folded in half to allow one drop of the thrombin solution and one drop of the fibrinogen solution to be applied and mixed. The graft was quickly placed on the setting fibrin clot and pressure was applied to facilitate adhesion. The same procedure was repeated for the remaining half of the graft. In the porous system, 16 mg of PEO was sprinkled on to the wound bed after the initial addition of thrombin, while 8 mg of PEO was added to each subsequent application of the thrombin solution.

#### 2.2.2. Necropsy

Sacrifice was performed at 3 and 10 days; 5 rabbits within each time period. A solution of ketamine at 30–44 mg/kg and xylazine at 5 mg/kg, was used to anesthetize the animal before injecting Socumb at 1 mL/10 Lbs., directly into the cardiac muscle. The healing wounds were excised and sectioned; half of each immediately fixed for 2–4 h with Pen–Fix (Richard Allen Medical Industries, Richland, MI, USA) with the remaining section placed in a 0.9% saline solution for preservation until mechanical testing was performed later in the day.

#### 2.2.3. Mechanical Testing

Mechanical testing was performed on an Instron Model 1114 (Instron, Canton, MA, USA) at the University of Alabama School of Dentistry. The excised grafts, after removing any muscle, were placed in the testing apparatus and strained at a rate of 0.5 cm/min. Length, width, and height of each section was recorded and later used to calculate the stiffness.

Porous and non-porous fibrin glues used in the study were also tested on the Instron. 1.9 cm × 1.0 cm sections of porcine skin were attached to thin strips of balsa wood. The glue (porous or non-porous) was applied on one section of skin, pressure was applied to simulate actual surgical conditions, and allowed to set for either 5 or 45 min. Three test strips of each type and time period were then strained at a rate of 5 cm/min. Shear strength of the glue was then calculated.

#### 2.2.4. Histology

Histological sections were obtained from the wounds fixed in 10% neutral buffered formalin and embedded in paraffin. Three levels within each wound, with six sections per level, were cut on a microtome (Leica, Wizlar, Germany). Before staining, all sections were deparaffinized and hydrated to water. Two sections in each level were stained with Gomori’s Trichrome and were used to obtain the volume fraction of new collagen in each wound. Two slides from each level were stained with Hemotoxylin and Eosin; the volume fraction of cells and blood vessel number and area fraction was then quantified.

#### 2.2.5. Microscopic Analysis

Data was obtained from tissue sections via histomorphometric techniques [33]. Nine fields per wound were analyzed with a 121-point grid (Nikon, Tokyo, Japan) to determine the volume percent of macrophages, neutrophils, and fibroblasts. Three different fields per slide were evaluated: within the fibrin glue and above (T1); below the fibrin glue (B1); and at the wound edge (S1). The above data was used with the relation: V = {(∑ P_f_)/(m × P_t_) × 100%}, where P_f_ is the number of grid points intercepted by the object of interest, m is the number of fields analyzed, and P_t_ is the total number of grid points to yield the desired information [33].

Measurements of blood vessel number and area fraction were carried out via an image processing system (IBAS). An image of the histological slide was created on a color monitor. After calibration, blood vessels were identified, and the perimeter circled in one of two areas: middle (*md*) and side (*sd*) of the T1 region. Automatic calculations gave perimeter and area within the enclosed area. Analysis included determining number per unit area and total blood vessel area in the measured field. The number of vessels per unit area approximates the length of blood vessels per unit volume assuming it is one long vessel where the blood vessel area fraction is a density measurement (approximates volume fraction) and is a way to compare relative angiogenic response [33].

#### 2.2.6. Statistics

Paired *t*-tests or multiple-comparison ANOVA and Tukey tests were performed to determine differences between treatments, time-periods, and fibrin glue strength. Calculations were made assuming α = 0.5.

### 2.3. Clinical Study

Eight patients were enrolled in a clinical study from the University of Alabama at Birmingham (UAB) Burn Center using the system optimized in the animal model. An Investigational New Drug Application (IND) was obtained from the FDA (Food and Drug Administration) for use of FGF-1 in this study. A non-porous Tissucol^®^ was used instead of making fibrin glue. The FGF-1 was added to the fibrinogen component as in the animal study.

Patients were given informed consent soon after admission to the UAB Burn Unit, at least 24 h before the scheduled burn graft surgery. For each patient, one full thickness graft site (relatively flat) of at least 3″ × 6″ (after excision of the eschar) was selected that could be divided into three similar sized areas of 3″ × 2″. As per the normal protocol, a donor site was selected, and the skin graft was harvested and meshed as done in the animal study (Zimmer Air Powered Dermatome (Model #8801-01, Zimmer Patient Care Division, Dover, OH, USA) and a 3:1 mesher (Brennan, North Bergen, NJ, USA). The meshed graft was cut to fit the burned area. There were two fibrin glue preparations (i.e., one with FGF-1 and one without) were applied to the wound beds on the two outer thirds of the wound, similar to the animal study, in a random blinded fashion. The middle third was stapled or sutured around the periphery, as per the surgeon’s normal protocol, to the wound bed.

For each of the three treatments per patient, two assessments were done at dressing changes over the first 50 days: blood flow and healing rate. Once the wounds had healed, mechanical testing was done in situ.

#### 2.3.1. Blood Flow

The middle portion of each of the three treatment areas were monitored for blood perfusion over the first 50 days post-surgery. A laser doppler scanner (Lisca, Hewitt, NJ, USA) using a He-Ne laser that scans across the wound, was used to detect blood perfusion without contacting the wound. A color map was produced with the highest flow depicted as red, and lowest as blue pixels. When the scanning head was situated approximately 10″ from the wound surface, each pixel represents 1 mm^2^. The values for each wound area were averaged together to get one value per treatment per time point.

#### 2.3.2. Healing Rate

Healing rate was measured from images taken of the wound (with a scale in the image as a reference). Again, the healing rate was determined from the middle portion of each wound area.

The healing rate or in this case epithelialization rate was calculated using a model proposed by Gilman [34] and later illustrated by Gorin et al. [35] to allow healing rate to be determined independent of wound size and shape. The method allows for the calculation of a linear healing rate perpendicular to [33,34,35,36,37] the wound edge as a function of wound area and perimeter: Healing rate = ∆ SA/(P_avg_ × ∆t), where ∆ SA is the change in surface area over the time period and P_avg_ is the average wound perimeter between initial and final time points (∆t). For these meshed skin grafts, the healing rate was calculated from the average of a minimum of four units of the mesh in the center of the graft.

#### 2.3.3. Mechanical Testing

The Biomechanical Tissue Characterization (BTC) system (BTC-2000, SRLI Technologies, Nashville, TN, USA) was used to measure tissue stiffness for time periods after the wound had healed. The BTC is a portable system consisting of a vacuum chamber and a computer. As a controlled load is applied to the tissue, tissue deflection is measured by a laser, and converted into a tissue stiffness measure. This gives a similar measure as the mechanical testing from the animal study.

#### 2.3.4. Data Analysis

For each test (i.e., healing rate, blood flow, and stiffness), measurements were made, as previously described, over a 50-day period. Due to UAB Burn clinic protocols, it was not possible to get measurements at the same time intervals for each patient. For each patient, the curves for the individual parameters were plotted over time for each of the three treatments. An average curve was made for each parameter using the data from all eight patients.

## 3. Results

### 3.1. Cell Count: 3-Day and 10-Day Time Periods

Results of the volume fraction of total cells, neutrophils, macrophages, and fibroblasts for the 3-day and 10-day time-periods are presented in Table 1, Table 2, Table 3 and Table 4. Each cell measurement category will be discussed in an individual section. Superscript text indicates a statistically significant difference between like symbols.

#### 3.1.1. Volume Fraction of All Cells (Tv)

Between the different types of treatments at three days for Tv in all regions of measurement, no statistical differences were detected. Likewise, when analyzed within the same treatment modality at different areas of counting, no statistical differences were noted. As with the 3-day data, no differences were noted between different types of treatment at the same level. There was also no statistical correlation within treatment type at different areas of measurement.

However, when 3-day data were compared with 10-day data, statistically significant increases (*p* < 0.05) in Tv were noted (60–190% increases). Differences at all levels were noted for treatments F and S; FW exhibited increases in T1 and S1, while PF and PFW showed increases in B1 and S1.

#### 3.1.2. Volume Fraction of Neutrophils (Nv)

Examination of 3-day data revealed statistical significance of Nv between treatment groups PFW, F, FW, and S in T1. For the PFW treatment (4.9 ± 2.6) notably increased (200–700%) compared to the other groups (except PF). No differences between treatment groups were discovered in other levels. Evaluation of data at different levels within a treatment type showed significant increase in Nv between T1 and both B1 and S1 (50–500%).

The 10-day data revealed no detectable differences between treatments at any level. There were also no significant results within treatment groups at different levels.

Comparison of Nv at 3 days and 10 days exhibited significant differences at all levels. Within the T1 region, all treatment groups showed significant decreases from 3 to 10 days (81%–96%). In the B1 group, only F was noted to have a detectable decrease (50%) in Nv. Finally, evaluation of region S1 indicated significant decreases for both FW and PFW (93% and 85% respectively).

#### 3.1.3. Volume Fraction of Macrophages (Mv)

The 3-day data analysis indicated significant increases of Mv in PFW (4.9 ± 3.3 with at least a 300% increase) relative to treatments F, FW, and S at level T1. Although no differences in Mv were detected in B1, significance was established in the S1 region between PFW (2.6 ± 1.1 with a 225% increase) and groups FW and S. There were no statistically significant differences noted within treatment regimens at different levels.

Evaluation of 10-day information revealed no statistical differences between treatments or at different levels within one treatment.

Comparison of Mv between 3 and 10 days indicated statistically significant decreases in two of the three levels. The T1 data showed significantly higher values for Mv at 3 days in the PF and PFW groups (150% and 490%, respectively). No statistical differences were noted in the B1 region; however, in the S1 region there was a statistically significant decrease (62%) of Mv in group PFW and an increase in macrophage number in treatment S (100%).

#### 3.1.4. Volume Fraction of Fibroblasts (Fv)

Data from the 3-day time-period indicated significant increases in the number of fibroblasts at two levels when comparing different treatments. In region T1, significant differences of Fv were noted between FW (3.2 ± 0.7) and PFW (1.0 ± 0.8). Level B1 showed no such differences, while in the S1 region there was a significant increase (175%) in F (4.9 ± 1.7) when compared to S (2.3 ± 0.4). For Fv, both PF and PFW were significantly increased in region B1 versus region T1 (115% and 440%, respectively).

Examination of 10-day Fv results revealed no statistically significant differences between treatments at any level. In addition, no significant differences were detected at different levels within a single treatment type.

For the 3-day and 10-day data comparison, a statistical increase in Fv was found at every level. Level T1 showed Fv increased in all treatments between days 3 and 10 (between 170% (FW) and 680% (PFW)). Level B1 exhibited statistically significant increases in F (114%), PF (97%), and S (190%). Region S1, like T1, revealed significant increases of Fv in every treatment type (between 110% (F) and 320% (S)).

### 3.2. Skin Graft Take

Subjective analysis of the skin grafts during healing and at necropsy (Table 5) revealed that take was more likely compromised in the PF and PFW groups (60% each at 3 days and 60% and 80%, respectively, at 10 days). All other grafts exhibited complete take at 3 days and only one FW graft showed no take at 10 days.

### 3.3. Blood Vessels

Calculation of the number of blood vessels per cm^2^ revealed no significant difference between treatments at either the 3-day or 10-day time-periods. However, there were significant differences between areas within the same treatment group at the two time-periods (Table 6). The PFW and S specimens in the *sd* region at three days showed 33.9 ± 27.0 bv/cm^2^ and 45.8 ± 22.6 bv/cm^2^, while at 10 days the results were 92.6 ± 40.7 bv/cm^2^ (170% increase) and 109.6 ± 34.0 bv/cm^2^ (140% increase), respectively. The F and S treatments showed significant results in the *md* region at the different time intervals (29.9 ± 17.4 bv/cm^2^ versus 57.0 ± 25.5 bv/cm^2^ (90% increase) and 29.9 ± 20.1 bv/cm^2^ and 98.6 ± 43.2 bv/cm^2^ (130% increase)).

Blood vessel volume fraction showed no difference between treatments at either time-period in the same region. There were, however, increases between the *sd* and *md* regions at three days (330% to 850%) as well as in the *sd* region between day 3 and day 10 (200% to 780%) (Table 7).

### 3.4. Mechanical Strength

Although mechanical testing between porous and non-porous fibrin glue preparations revealed no significant differences of shear strength at 5 min or 45 min, there was a 45 to 68% increase (Table 8). Likewise, there was no detected difference between the formulations at the two time intervals.

Testing of samples obtained from each of the treatment regimens showed no significant differences in elastic modulus within a treatment at 3 days or 10 days (Table 9). The only significant finding was that between the S and FW (101.5 ± 41.8 and 34.4 ± 15.7 about a 200% increase) treatments during the 3-day time-period. The increase between S and PF was 300% but was not a statistically significant difference. Note the NL is normal skin and therefore only one time point.

### 3.5. Clinical Study

**Figure 1:**
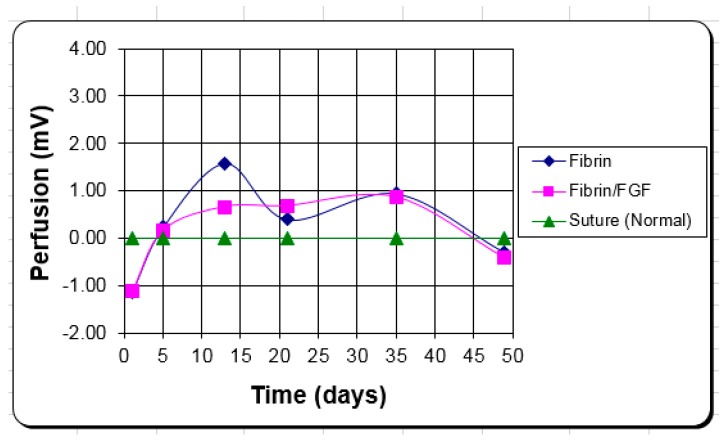
The average perfusion data for the clinical burn patients. The two fibrin treatments are scored relative to the sutured control.

The average curve for blood perfusion of the three treatments is shown in Figure 1. This graph shows the change in perfusion level of the fibrin systems compared to the control. On average, both fibrin systems appear to increase blood flow over the controls. For the first five days, however, the controls are higher, but the FTAs are higher for the next 40 days with complete epithelialization by 21 days. The variation of the curves of the eight patients at any given point would be similar to a standard deviation of approximately ¼–1/3 of the mean. This means that 68% of the perfusion levels would be ±¼–1/3 of the value on the curve.

**Figure 2:**
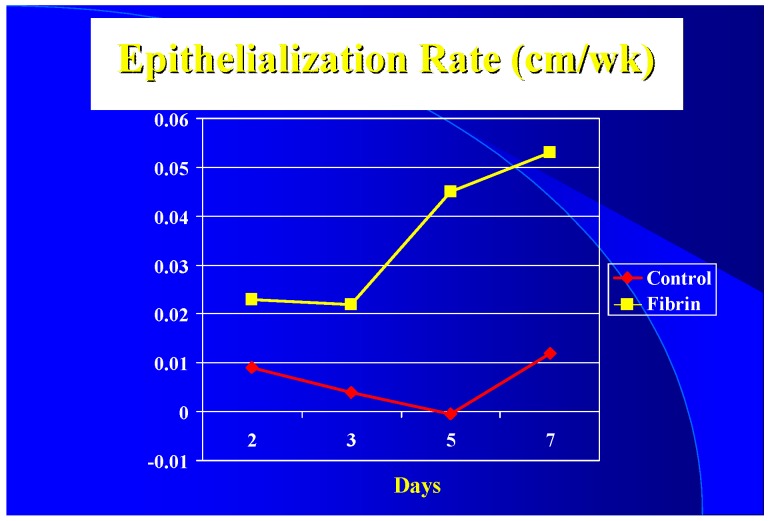
The average epithelialization rate (cm/wk) of the FTA and controls for the first seven days.

For epithelialization rates, the average curves were elevated for both fibrin treatments (both were similar enough to be grouped here), with an apparent lag time (5 days for the control) leading to a decrease in healing time of 5 days (21 versus 16 days) (Figure 2). Again, the variation of the curves of the eight patients at any given point would be similar to a standard deviation of approximately ¼–1/3 of the mean.

**Figure 3:**
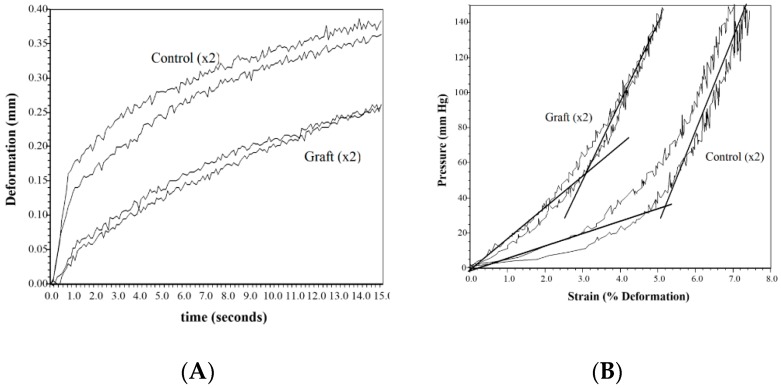
Representative graphs of the stiffness of the treated graft vs. the control. (**A**) Deformation vs. time, and (**B**) pressure vs. strain.

The tissue stiffness difference was comparable to the animal study although the assessments were done after a month when complete healing was achieved (Figure 3). The controls were about twice as stiff and required longer rehabilitation time. Both graphs show the relative stiffness between the treated graft and the control. The first also shows the time dependent response or viscoelastic response. The second is the more typical stress vs. strain plot although the stress in this case is from a vacuum. This is a representative curve that is close to the average of the eight patients.

## 4. Discussion

Reducing healthcare costs is an important goal. It is complicated by the fact that healthcare costs are driven by multiple factors, as well as that the cost is shared between patients, providers, and insurance companies. Further, it is not always clear how much of a cost reduction is worthwhile, especially if they is a resultant reduction in the quality of care. This study, however, was looking at the feasibility of reducing healthcare costs while still improving the clinical performance.

Specifically, to increase the clinical performance of the standard of care for full-thickness burns (meshed skin graft) by speeding the healing rate in order to make a significant difference in scar formation. This should translate to improvement in both cosmetic and functional clinical performance as well as reduce the rehabilitation time. This would significantly reduce the $75,000 average per burn patient in the hospital, as well as costs for rehabilitation [4]. The increased cost of the treatment, however, has to be considered. Adding in fibrin glue would not significantly alter the cost of treatment. Use of a growth factor, however, could significantly increase the cost of the procedure due primarily to the increased development costs of the adhesive system. In this case, therefore, the value added by including the growth factor has to be weighed against the added cost of the treatment. 

Although the angiogenic growth factor (FGF-1) added to a FTA (fibrin tissue adhesive) in open wounds, made a significant impact on healing [31,32], it did not appear to do so when used with meshed skin grafts. This is probably due to the difference in function of the FTA between the two applications. In open wounds, the FTA is the scaffold system for the entire wound. Formation of tissue requires an angiogenic response to provide the fibroblasts enough oxygen to produce the extracellular matrix to replace the degrading scaffold starting from the edge of the wound [7,8,9,24]. In a meshed graft, the fibrin is needed to facilitate the attachment and/or ingrowth of blood vessels from the wound bed to the graft [28,29]. Therefore, in the meshed skin graft systems evaluated, it appears that the insignificant benefit of using the growth factor would not be worth the additional cost of the treatment.

Specifically, the study was designed to use these systems in both an animal model and clinically. The intent was to not only quantify the benefit for clinical performance, but also quantify the changes in bioprocesses to help determine the mechanisms for any benefits seen. In the animal model both (healing rate and bioprocess rate) can be looked at with only clinical performance (plus angiogenic response) easily studied clinically. Fibrin tissue adhesives have been successfully used in many areas of clinical medicine, including delivering medications as well as growth factors, to obtain clinical benefit [12,24,28,29]. Previous studies have shown that it is possible to create a porous fibrin scaffold with time-dependent delivery of FGF-1 [31,32], but no data exists on its clinical use in conjunction with skin grafting.

The cellular data, in this study, showed that although no statistically significant difference was noted in Tv (total cell volume fraction), several individual cell populations showed differences for certain treatment types at different time periods. Both the PF and PFW (porous fibrin with and without FGF-1) groups attracted neutrophils in greater numbers than other treatments, predominantly in the T1 region, where the FTA was applied. Although the PEO, used to make pores, hydrolyzes to a chemically inert compound and is water soluble, the large molecular weight of the polymer may interfere with rapid hydrolysis to smaller components. It is therefore possible that the PEO affects the wound as an irritant and disrupts wound healing. As with any traumatic wound, it must be free of debris for rapid tissue ingrowth. This was corroborated by the fact that PF and PFW treated wounds tended to have decreased graft take as well as an increase in the macrophage response. This again could be due to the macrophages required to phagocytize the PEO particles. Although this increase in PF and PFW treated wounds occurred at 3 days, the 10-day data revealed no significant differences between treatments in Nv or Mv as well as an overall decrease in cells compared to the 3-day data. This suggests that even though the PEO may act as an irritant early in wound healing, by day 10 this may have subsided along with a reduction in the overall inflammatory response.

For Fv (fibroblast volume fraction), as expected, there was a more vigorous response with FGF-1 particularly in T1 at 3 Days, consistent with previous studies involving FGF-1 and fibroblast activity [24,31]. The porous systems, with FGF-1 (PFW), however, did not show an increase in fibroblast response; probably due to the increased infiltration of inflammatory cells and the delay of wound healing.

The function of the FGF-1 added to the FTA was to provide additional angiogenic stimulus for more rapid and successful graft take. Analysis of the data revealed no statistical difference in the number of blood vessels or volume fraction of blood vessels between any of the treatments as well as no clinical difference in the angiogenic response (blood perfusion). There were differences between time points in the same region (number of vessels) and between regions at the same time period (volume fraction of vessels) but no statistical differences between with or with FGF-1, or even consistent trends.

Again, the difference in the fibrin function from an open wound scaffold to an adhesive for a skin graft could help explain why FGF-1 appeared to be effective in one case but not the other. It is probable that the activity level over time of the FGF-1 is different between the two applications due to different release profiles or different half-lives between the two environments; but most likely not enough to make a significant difference. It is more likely that the thinner profile of the fibrin as a tissue adhesive vs. an open wound scaffold makes the difference. For skin grafts, the proximity of the graft to the wound bed is important [4,8,24], and the fibrin alone seems to have a sufficient angiogenic response over the short distance to make the FGF-1 unnecessary.

The clinical blood perfusion level is closer to the in vivo volume fraction of blood vessels measure than the number per area. Again, the better attachment to the wound bed by the fibrin is what appears to provide the clinical advantage vs. the angiogenic response of FGF-1. The fibrin alone keeps the blood perfusion level up to improve healing with little to no added benefit from the FGF-1, in this case.

Inadequate mechanical strength, however, may have affected the ability for angiogenesis to lead to skin graft take, in this study, making the porous systems not as successful as anticipated. Testing revealed, as expected, an inverse relationship between porosity and strength (decreasing strength with increasing porosity). The differences (30–40% decreases). however, were not statistically different most likely due to the small sample (*n* = 3) and high standard deviation (25–45% of the mean). If the strength becomes a clinical issue (graft stabilization), the strength of the FTA can be increased by increasing the fibrinogen concentration or slowing the degradation with an anti-fibrolytic agent, like aprotinin, or a cross-liking agent like Factor XIII [29,38,39].

Stiffness and scarring of the wound are a concern for both the patent and the physician. The elastic modulus measures the distensibility of the tissue and is the critical clinical measure that is worked on to get close to normal skin. Although not statistically different from the normal control, the sutured wound showed a trend towards increased modulus at both 3 days and 10 days (80–110% increase). The differences also were not statistically different most likely due to the small sample (*n* = 5) and high standard deviation (40% of the mean at 3 days and over 100% at 10 days). The treated wounds were close to the normal skin values versus twice the values for the sutured control. Only the FW at day 3 was statistically different. The clinical data showed this same trend with the controls about twice the stiffness of the treated samples even after a month of healing, supported by other studies [24,26,29,37]. This clinically manifests itself as the anticipated scar tissue, which can complicate function and appearance [24,29]. If the treated wounds have a stiffness comparable to normal skin vs. the twice as stiff controls (Figure 3), the one- to two-year rehabilitation time to get flexibility back to normal can be reduced if not eliminated, leading to both a reduction in cost and a clinical improvement [24].

The animal study looked at graft take as the healing performance measure, with the clinical study looking at epithelialization rate. The short-term in vivo study showed that the porous fibrin systems seemed to perform the worst, probably due to the increased inflammatory response and mechanical instability negating any benefit of using a porous system. 

Clinically the epithelialization rate was used vs. graft take. It appears that with a 3 to 1 mesh graft that the controls (sutured or stapled) had at least a five-day lag in epithelialization over the fibrin treated grafts (Figure 2) resulting in a five-day difference in closure time (16 versus 21 days). Due to treatment protocols, it was not possible to get epithelialization rates after seven days. It appears, however, once started that the rate increases until it peaks at the 0.1–0.2 cm/wk level seen by others [40].

In both the animal and clinical studies, the “n” number was too low and standard deviations too high to allow the tests to be sensitive enough to pick up all the differences that would make a clinical difference. It normally required an increase of over 100% to be detected. In some cases, however, ANOVA was able to pick up these differences not statistically significant in the multiple comparisons tests. These include Fv in S1 at 10 days, Mv in T1 at 3 days, and Nv within PF at 3 days. For the *t*-tests, increasing the sample size most likely would result in more of the clinically significant differences being statistically different.

Although not all differences were statistically significant, the results suggest the following:

The use of FTAs on a meshed skin graft appears to speed up the regenerative healing rate leading to less scarring in the holes in the mesh. It appears to shorten the healing time by five days and keeps the tissue stiffness close to normal levels vs. the doubling of the stiffness by the controls. Although making the fibrin porous and adding an angiogenic agent were both supposed to increase the healing rate further, neither seemed to make a significant clinical difference and the porous systems tested had reduced graft take.

It appears that the FTA performed better than the controls, serving as an adhesive and a scaffold. The fibrin allows closer and more stable attachment of the graft to the wound bed, as well as serves as a scaffold for the blood vessels to grow through to reattach to the graft. Histologically the controls had more of an inflammatory response (macrophage volume fraction) and less of a healing response (fibroblast volume fraction) compared to the FTAs in the first ten days. Although there were not statistically significant differences, among treatments, for the volume fraction of blood vessels during the first 10 days, the Laser Doppler scanner showed that for all time points after five days, the blood perfusion was greater in the FTAs than the controls.

It appears that although graft take was not much different by ten days, the healing in the spaces of the mesh started earlier in the FTAs. Epithelialization seemed to have a five-day lag in the controls compared to the FTAs. It is possible that for the first five days the blood perfusion response in the controls was more due to inflammation than healing; after that, the elevated blood flow in the FTAs probably helped keep the healing rate higher than the controls.

Healing does not usually start until after the inflammatory phase starts to resolve. Both better attachment of the mesh to the wound base and the use of the fibrin as a dermal scaffold could have led to a faster resolution of the inflammatory stage as well as the observed quicker start to re-epithelialization. The quicker start to healing led to a faster closure of the wound, and therefore, less scarring. Therefore, the FTAs can allow wound closure about five days earlier than the controls leading to a better cosmetic outcome. The earlier wound closure would reduce the time for wound dressings, as well as reduce the chance of infection. Having the resultant wound less stiff would lead to a shortened rehabilitation time. Therefore, using fibrin alone appears to meet the goals of the study: reducing scarring in meshed skin graft healing leading to a better cosmetic result and reducing the cost and time of rehabilitation to restore function. The fibrin appears to do this by increasing the closure rate of a meshed skin graft to reduce scarring, which should also decrease the infection rate.

Although it appears that the fibrin system provides a better result clinically from both a cost and performance perspective, the sample size needs to be increased to prove this benefit as well as the mechanism for this improvement. A five-day decrease in closure time and a reduction in stiffness close to normal values (vs. twice the stiffness for controls) both are significant clinical improvements. Eliminating the need to look further at porous systems or FGF-1 delivery to get the desired clinical result, will streamline future in vivo and clinical studies. This will also make it easier to determine if the proposed mechanisms are further supported in larger in vivo and clinical studies.

## Figures and Tables

**Table 1:** Volume percentage of cells at 3 days and 10 days grouped by treatment for each area of the wound.

T1		
	**3-Day**	**10-Day**
**F**	6.7 ± 2.0 *	18.5 ± 4.2 *
**FW**	6.5 ± 1.4 ^@^	15.5 ± 2.9 ^@^
**PF**	10.4 ± 4.1	15.5 ± 2.9
**PFW**	10.7 ± 5.6	17.7 ± 3.4
**S**	6.0 ± 3.3 ^+^	16.5 ± 3.7 ^+^
**B1**		
	**3-Day**	**10-Day**
**F**	7.1 ± 1.8 *	16.6 ± 3.8 *
**FW**	7.5 ± 4.1	11.5 ± 1.4
**PF**	9.1 ± 4.2 *	14.5 ± 3.7 ^$^
**PFW**	8.2 ± 3.2 ^&^	13.7 ± 1.6 ^&^
**S**	4.7 ± 2.3 ^+^	14.0 ± 2.6 ^+^
**S1**		
	**3-Day**	**10-Day**
**F**	7.8 ± 2.9 *	17.2 ± 3.0 *
**FW**	5.3 ± 1.0 ^@^	14.7 ± 2.0 ^@^
**PF**	5.8 ± 2.3 ^$^	16.2 ± 2.5 ^$^
**PFW**	7.2 ± 2.1 ^&^	14.9 ± 1.6 ^&^
**S**	4.5 ± 2.4 ^+^	15.1 ± 3.6 ^+^

**Table 2:** Volume percentage of neutrophils at 3 days and 10 days grouped by treatment for each area of the wound.

T1		
	**3-Day**	**10-Day**
**F**	1.2 ± 1.0 *	0.1 ± 0.2 *
**FW**	1.6 ± 1.4 ^@^	0.3 ± 0.3 ^@^
**PF**	3.8 ± 1.7 ^$^	0.4 ± 0.5 ^$^
**PFW**	4.9 ± 2.6 ^&^	0.2 ± 0.2 ^&^
**S**	0.6 ± 0.5 ^+^	0.05 ± 0.1 ^+^
**B1**		
	**3-Day**	**10-Day**
**F**	0.8 ± 0.7 *	0.4 ± 0.2 *
**FW**	0.9 ± 1.0	0.3 ± 0.5
**PF**	1.6 ± 1.0	1.4 ± 1.4
**PFW**	1.9 ± 1.9	1.0 ± 0.8
**S**	0.5 ± 0.4	0.1 ± 0.1
**S1**		
	**3-Day**	**10-Day**
**F**	0.8 ± 1.0	0.05 ± 0.1
**FW**	0.7 ± 0.4 ^@^	0.05 ± 0.1 ^@^
**PF**	1.0 ± 0.8	0.2 ± 0.2
**PFW**	1.2 ± 0.4 ^&^	0.06 ± 0.1 ^&^
**S**	0.1 ± 0.2	0.1 ± 0.2

**Table 3:** Volume percentage of macrophages at 3 days and 10 days grouped by treatment for each area of the wound.

T1		
	**3-Day**	**10-Day**
**F**	1.0 ± 0.3	0.9± 0.6
**FW**	1.1 ± 0.6	1.0 ± 0.7
**PF**	3.3 ± 1.1 ^$^	1.3 ± 1.0 ^$^
**PFW**	4.7 ± 3.3 ^&^	0.8 ± 0.6 ^&^
**S**	0.8 ± 1.5	0.7 ± 0.5
**B1**		
	**3-Day**	**10-Day**
**F**	1.3 ± 0.4	0.8 ± 0.7
**FW**	1.2 ± 1.0	0.9 ± 1.0
**PF**	2.5 ± 1.4	1.6 ± 1.0
**PFW**	2.2 ± 1.1	1.9 ± 1.9
**S**	0.6 ± 0.3	0.5 ± 0.4
**S1**		
	**3-Day**	**10-Day**
**F**	1.7 ± 1.0	1.2 ± 1.2
**FW**	0.8 ± 0.8	0.8 ± 0.7
**PF**	1.6 ± 0.9	0.9 ± 0.6
**PFW**	± 1.1 ^&^	1.0 ± 1.1 ^&^
**S**	0.8 ± 0.3 ^+^	1.6 ± 0.2 ^+^

**Table 4:** Volume percentage of fibroblasts at 3 days and 10 days grouped by treatment for each area of the wound.

T1		
	**3-Day**	**10-Day**
**F**	2.8 ± 1.2 *	9.0 ± 1.4 *
**FW**	3.2 ± 0.7 ^@^	8.6 ± 2.0 ^@^
**PF**	2.1 ± 0.9 ^$^	8.1 ± 2.1 ^$^
**PFW**	1.0 ± 0.8 ^&^	7.8 ± 2.2 ^&^
**S**	1.4 ± 1.0 ^+^	9.3 ± 0.3 ^+^
**B1**		
	**3-Day**	**10-Day**
**F**	4.6 ± 2.4 *	10.3 ± 1.6 *
**FW**	5.2 ± 2.4	8.9 ± 3.7
**PF**	4.7 ± 1.6 ^$^	9.3 ± 2.1 ^$^
**PFW**	5.4 ± 2.9	8.2 ± 1.9
**S**	3.2 ± 1.9 ^+^	9.4 ± 1.1 ^+^
**S1**		
	**3-Day**	**10-Day**
**F**	4.9 ± 1.7 *	10.0 ± 1.4 *
**FW**	3.2 ± 1.2 ^@^	7.9 ± 0.9 ^@^
**PF**	3.2 ± 0.7 ^$^	9.7 ± 1.7 ^$^
**PFW**	2.7 ± 1.1 ^&^	7.6 ± 1.2 ^&^
**S**	2.3 ± 0.4 ^+^	9.6 ± 1.5 ^+^

**Table 5:** Percentage of viable grafts in each time period.

	3-Day	10-Day
**F**	100	100
**FW**	100	80
**PF**	60	60
**PFW**	60	80
**S**	100	100

**Table 6:** Number of blood vessels per area (#/cm^2^).

		3 Day-*sd*		10 Day-*sd*		3 Day-*md*		10 Day-*md*
**F**	*N* = 5	37.1 ± 23.7	*N* = 4	101.6 ± 98.0	*N* = 5	29.9 ± 17.4 ^#^	*N* = 5	57.0 ± 25.5 ^#^
**FW**	*N* = 5	56.2 ± 37.0	*N* = 5	65.8 ± 16.9	*N* = 5	55.0 ± 31.5	*N* = 5	63.4 ± 19.2
**PF**	*N* = 4	71.8 ± 44.5	*N* = 5	71.7 ± 31.9	*N* = 4	52.3 ± 32.9	*N* = 5	138.7 ± 94.1
**PFW**	*N* = 3	33.9 ± 27.0 ^@^	*N* = 4	92.6 ± 40.7 ^@^	*N* = 4	52.3 ± 37.3	*N* = 4	85.2 ± 44.6
**S**	*N* = 3	45.8 ± 22.6 *	*N* = 3	109.6 ± 34.0 *	*N* = 4	29.9 ± 20.1 ^$^	*N* = 4	98.6 ± 43.2 ^$^

Note: superscript text indicates a statistically significant difference between like symbols.

**Table 7:** Blood vessel volume fraction (%).

		3 Day-*sd*		10 Day-*sd*		3 Day-*md*		10 Day-*md*
**F**	*N* = 5	0.072 ± 0.07 ^a^	*N* = 4	0.22 ± 0.13 ^a^	*N* = 5	0.20 ± 0.15	*N* = 5	0.15 ± 0.07
**FW**	*N* = 5	0.076 ± 0.04 ^c^	*N* = 5	0.16 ± 0.05 ^c^	*N* = 5	0.44 ± 0.39	*N* = 5	0.16 ± 0.05
**PF**	*N* = 4	0.059 ± 0.03 ^e^	*N* = 5	0.24 ± 0.16 ^e^	*N* = 4	0.35 ± 0.21	*N* = 5	0.32 ± 0.16
**PFW**	*N* = 3	0.037 ± 0.03 ^i^	*N* = 4	0.28 ± 0.17 ^i^	*N* = 4	0.15 ± 0.10	*N* = 4	0.36 ± 0.23
**S**	*N* = 3	0.042 ± 0.04 ^r^	*N* = 3	0.37 ± 0.12 ^r^	*N* = 3	0.40 ± 0.34	*N* = 4	0.31 ± 0.20

Note: superscript text indicates a statistically significant difference between like symbols.

**Table 8:** Peak shear force, porous vs. non-porous fibrin glue (N).

		Porous		Non-Porous
**5 min**	*N* = 3	0.99 ± 0.37	*N* = 3	1.67 ± 0.60
**45 min**	*N* = 3	1.59 ± 0.25	*N* = 3	2.29 ± 1.06

**Table 9:** Elastic modulus.

		3 Day		10 Day
**F**	*N* = 5	63.0 ± 28.5	*N* = 5	57.4 ± 43.9
**FW**	*N* = 5	34.4 ± 15.7 *	*N* = 5	51.6 ± 29.6
**PF**	*N* = 4	46.34 ± 32.1	*N* = 5	30.5 ± 21.6
**PFW**	*N* = 5	54.1 ± 32.1	*N* = 5	54.4 ± 35.2
**S**	*N* = 5	101.5 ± 41.8 *	*N* = 5	123.3 ± 142.5
**NL**	*N* = 3	56.0 ± 23.9	*N* = 5	

Note: superscript text indicates a statistically significant difference between like symbols.
